# The risk of clinically diagnosed gout by serum urate levels: results from 30 years follow-up of the Malmö Preventive Project cohort in southern Sweden

**DOI:** 10.1186/s13075-018-1697-6

**Published:** 2018-08-29

**Authors:** Meliha C. Kapetanovic, Peter Nilsson, Carl Turesson, Martin Englund, Nicola Dalbeth, Lennart Jacobsson

**Affiliations:** 1 Department of Clinical Sciences Lund, Section of Rheumatology, Lund University and Skåne University Hospital, Kioskgatan 5, SE-221 85 Lund, Sweden; 2 Department of Clinical Sciences, Lund University, Malmö, Sweden; 3 Department of Emergency and Internal Medicine, Skåne University Hospital, Malmö, Sweden; 4 Department of Clinical Sciences, Malmö, Lunds University and Skåne University Hospital, Malmö, Sweden; 5 Clinical Epidemiology Unit, Orthopaedics, Department of Clinical Sciences, Lund University, Lund, Sweden; 6 Department of Medicine, University of Auckland, Auckland, New Zealand; 7 Department of Rheumatology and Inflammation Research, Sahlgrenska Academy at University of Gothenburg, Gothenburg, Sweden

**Keywords:** Hyperuricemia, Incident gout, Risk factors

## Abstract

**Background:**

Hyperuricemia (HU) is in the causal pathway for developing clinical gout. There are few population-based assessments of the absolute and relative risk of clinically diagnosed incident gout in subjects with HU. We aimed to explore the long-term risk of developing incident gout among asymptomatic adults with different levels of serum urate (SU).

**Methods:**

Malmö Preventive Project was a population-based screening program for cardiovascular risk factors, alcohol abuse, and breast cancer in Malmö, Sweden. The study population was screened between 1974 and 1992. At baseline, subjects were assessed with a questionnaire, physical examination, and laboratory tests. Follow-up ended at first gout diagnosis, death, moving from area, or December 31, 2014. Incident gout (using ICD10 codes) was diagnosed based on national registers for specialized inpatient and outpatient care, and from 1998 onward in the Skåne Healthcare Register including primary healthcare. Incidence rates, absolute risk, hazard ratios (HRs) and potentially associated factors were analyzed by baseline SU levels, i.e. normal levels (≤ 360 μmol/L); 361–405 (levels below tissue solubility of SU), and > 405 (HU), overall, and by sex.

**Results:**

Overall, 1275 individuals [3.8%; 1014 men (4.5%) and 261 women (2.4%)] of the 33,346 study participants (mean age: 45.7 (SD: 7.4), 67% men), developed incident gout during follow-up (mean 28.2 years). Of those with HU, 14.7% of men and 19.5% of women developed gout. Compared to subjects in the lowest SU category, the age-adjusted HR in men increased from 2.7 to 6.4, and in women from 4.4 to 13.1 with increasing baseline SU category, and with a statistically significant interaction of sex (*p* < 0.001). Body mass index, estimated glomerular filtration rate (negative), triglycerides, alcohol risk behavior (only in men), and comorbidities such as hypertension, cardiovascular disease, and diabetes were strongly associated with SU at baseline in both sexes.

**Conclusions:**

The absolute risk for developing clinically diagnosed gout over 30 years in middle-aged subjects was 3.8%, and increased progressively in both men and women in relation to baseline SU. This risk increase was significantly higher in women than in men, whereas the associations between baseline risk markers and SU levels were similar in both sexes.

## Background

Hyperuricemia (HU) is defined as serum urate concentrations above its levels of solubility in the serum [1–9], which is considered to be at approximately 405 mmol/L (6.8 mg/dL) [10]. We have previously reported a prevalence of 1.7% for clinically diagnosed gout among adults in two regions in Sweden [11, 12], but there are limited data regarding the risk of incident gout in individuals with asymptomatic HU [9]. Asymptomatic HU is common and, depending on the chosen cutoff value, figures of 10–20% have been reported in Western populations [1–3]. Unpublished data from the ongoing Swedish CArdioPulmonary BioImage Study (SCAPIS) in Sweden suggest that 20% of men and 12% of women with mean age 57 years have HU (personal communication, Mats Dehlin). A systematic review and meta-analysis of studies conducted in China reported pooled prevalence of HU and gout being 13.3% and 1.1%, respectively [7]. HU and gout have repeatedly been reported to be associated with the metabolic syndrome, several comorbidities, and unhealthy lifestyle factors in non-European and European populations [13]. It is commonly referenced that 10–20% of subject with HU will develop clinically relevant gout, the most established adverse health outcome of HU [1, 5, 6, 9], whereas it still debated whether HU is causally related to other chronic diseases such as cardiovascular disease (CVD), kidney disease, dementia, or cancer [13], or not. At present, asymptomatic HU is therefore not considered as an indication for urate-lowering therapy (ULT) [14]. The risk of incident gout has been reported to increase with duration of HU and degree of increased SU levels [1, 3, 4, 7–9, 13, 15]. These estimates are all based on population outside Europe and often on relatively small samples [1, 3, 4, 7]. Population-based studies from northern Europe are lacking.

Thus, the aims of the present study were: (1) to determine the long-term absolute and relative risks of incident clinically diagnosed gout among adults without gout by different levels of baseline HU; and (2) to describe the relation between SU levels and potentially associated factors. For the analyses we used participants in the Malmö Preventive Project (MPP), a large-scale population based screening and case-finding program for cardiovascular risk factors, alcohol abuse, and breast cancer in Sweden.

## Methods

### Malmö Preventive Project (MPP)

#### Setting

In the city of Malmö, a screening program for cardiovascular risk factors, alcohol abuse, and breast cancer was started in 1974 at the Department of Preventive Medicine, University Hospital, Malmö, Sweden [16]. At the study start, the city population comprised approximately 250,000 inhabitants. The aim of MPP was to screen large strata of the adult population in order to find high-risk individuals for preventive interventions directed against cardiovascular risk and alcohol abuse. No specific intervention was offered to subjects with HU.

A total of 33,346 subjects participated in the health survey and their data constitute baseline information. The study population was screened between 1974 and 1992, with a total of 22,444 men and 10,902 women attending. The overall attendance was 71% (range 64–78% for different years). Men were mostly screened in the first half of the period (1974–1982), and women in the latter half (1981–1992), thus with different length of follow-up time. The main results of this survey were described previously [16].

#### Baseline information from survey

Subjects were invited to participate in a health screening which included: a self-administered questionnaire with 260 questions gathering information on socioeconomic factors, history of CVD, hypertension and diabetes mellitus (DM) (including family history for these conditions), smoking habits, alcohol consumption behavior, physical activity at work and during leisure time, dietary habits and weight gain, presence of signs and symptoms of CVD and history of malignancies); a physical examination (including weight, height, body mass index (BMI), and blood pressure), and laboratory testing (including serum urate level [SU], s-cholesterol; fs-triglycerides, s-creatinine, fasting blood glucose).

Estimated glomerular filtration rate (eGFR) was calculated using the Malmö-Lund revised equation based on the Swedish population [17].

Alcohol consumption behavior was studied using the Malmö modified brief Michigan Alcohol Screening Test (Mm-MAST) comprising nine questions regarding alcohol habits [18, 19]. These were: “Do you take a drink before going to a party?”, “Do you usually drink a bottle of wine or corresponding amounts of alcohol over the weekend?”, “Do you drink a couple of drinks (beers) a day to relax?”, “Do you tolerate more alcohol now than you did 10 years ago?”, “Have you difficulties not drinking more than your friends?”, “Do you fall asleep after moderate drinking without knowing how you got to bed”, “Do you have a bad consciousness after a party?”, “Do you take a drink (a beer) after the party?” and “Do you try to avoid alcoholic beverage for a determined period of time e.g. a week?” Confirming answers to at least two of these questions was used to identify the alcohol risk consumption behavior in the present study according to previous studies [18–20]. Mm-MAST could not be analyzed for women due the high frequency of missing data.

A question regarding history of gout was included in the questionnaire. In the present study, concomitant CVD (yes/no) at baseline was defined as history of angina pectoris, myocardial infarction (MI) or concomitant medication for heart disease, and DM at baseline (yes/no) as fasting blood glucose ≥6.7 mmol/L (in blood, according to contemporary reference levels in the 1970s) or history of DM. All individuals with a systolic blood pressure ≥ 160 mmHg at baseline, or those who reported being on antihypertensive treatment, were considered to have hypertension.

Data on marital status, retrieved from the Swedish population and housing census were categorized in five categories: “unmarried”, “married”, “divorced”, “widow/widower”, and “not specified” [21].

#### Identification of incident gout and follow-up

In order to identify all gout diagnoses (using International Classification of Diseases version 10 [ICD10] codes) given at visits to physicians within primary care (from 1998), specialized inpatient (from 1974) and outpatient specialized care (from 2001) the MPP cohort was linked to regional healthcare register (The Skåne Healthcare Register; SHR) and to National Patient Register (NPR), respectively.

Follow-up started at the time point of baseline screening that could vary between 1973 and 1992. Participants were followed until date of first gout diagnosis, death, moving from area, or December 31, 2014. Since the method for identifying incident gout was based on inpatient care up to 1998 and all types of care thereafter (including primary care), results are presented also for the two time periods separately; screening up through 1997 (or end of follow-up if before) and from 1998 until end of follow-up.

Baseline SU levels were stratified into three categories: i.e. normal levels (≤ 360; 361–405 (levels below tissue solubility of UA) and > 405 μmol/L, based on the conception that urate saturation occurs at levels > 405 μmol/L at physiological pH and body temperature and that levels ≤360 μmol/L is the treatment target for ULT [10]. HU was defined as SU > 405 μmol/L.

### Statistical analysis

Pearson correlation, Mann-Whitney *U* test and chi-squared test were used to analyze the associations between, on one hand, comorbidities, socioeconomic and lifestyle-related factors, with SU levels and HU on the other.

Cox regression models, stratified by sex, were applied to determine the hazard ratio (HR), with 95% confidence intervals (95% CI) of incident gout during the two time periods, i.e. date of baseline screening through December 31, 1998, and from January 1, 1999 through December 31, 2014; unadjusted and age-adjusted. The proportional hazard (PH) assumptions for Cox regression analysis were evaluated graphically in survival curves and log-minus-log plots and were found to be valid for the two time periods separately. Interactions between SU category and sex were tested in models separately for the time periods that included all subjects.

Incidence rates of gout in patients with different SU levels were calculated separately for men and women, both for the whole period of follow-up and stratified for the two time periods. Incidence was computed as number of individuals with first-time diagnosis of gout divided by the sum of follow-up time (person-years at risk) by sex and SU level category. A Poisson distribution was assumed when estimating 95% CIs for incidence rates. All analyses were performed by use of IBM SPSS Statistics 24 (IBM Corp., Armonk, NY, USA).

### Ethical considerations

The ethical approval was obtained from the Regional Ethics Committee at Lund University (Dnr 85/2004).

## Results

### Study participants

In total, 33,346 individuals (67% men, mean age 45.7 years at inclusion, mean follow-up 28.2 years; SD 8.4 years) participated in the MPP, and contributed overall with 936,826 person-years at risk (men: 647,114 person-years, women: 289,712 person-years). Patients with gout prior to inclusion (*n* = 11) were excluded from the analysis.

Table 1 summarizes the baseline characteristics of all participants MPP, stratified by sex.

**Table 1:** Baseline characteristics of participants in the Malmö Preventive Project (MPP) stratified by sex

	All	Men	Women
Sex, men n (%)	33,335	22,433 (67.3)	10,902 (32.7)
Age* (years)	45.7 (7.4)	43.7 (6.6)	49.7 (7.4)
BMI (kg/m^2^)	24.6 (3.6)	24.7 (3.3)	24.4 (4.2)
Serum urate (micromol/L)	300.8 (70.1)	323.2 (64.2)	254.4 (57.8)
Seum urate > 405, n (%)	2455 (7.1%)	2191 (9.8%)	164 (1.5%)
SBP in individuals with non-treated hypertension at baseline (mmHg)**	149 (21)	150 (20)	148 (21)
Fasting glucose (millimol/L) in non-DM individuals at baseline	4.9 (0.5)	4.9 (0.5)	4.8(0.5)
eGFR (ml/min/1.73m^2^)	77.9 (10.8)	79.1 (10.5)	75.6 (10.9)
Cholesterol (milimol/L)	5.7 (1.1)	5.6 (1.1)	5.8 (1.1)
Triglycerides (milimol/L)	1.4 (0.9)	1.5 (1.1)	1.1(0.6)
Hypertension at baseline n (%)^§^	6070 (17.2)	3672 (16.4)	2398 (22)
Diabetes mellitus at baseline, n (%)^§§^	828 (2.5)	575 (2.6)	253 (2.3)
Family history of any sibling having hypertension, n (%)	2519 (7.6)	1102 (4.9)	1417 (13)
History of kidney stone attack, n (%)	2729 (8.2)	2003 (8.9)	726 (6.7)
History of several kidney stone attacks, n (%)	1359 (4.1)	1046 (4.7)	313 (2.9)
Mm-MAST ≥ 2, n (%)^#^	21.7	30.8	2.8
ESR (mm/hour)	7.1 (6.9)	5.7 (6.0)	9.9 (7.7)
Current smoker, n (%)	14,846 (44.5)	11,038 (49.2)	3808 (37.1)

*Values for continuous variables denote mean (SD)

**Number of patients are 2780 male and 1416 female)

^#^Number of subjects are 6920 (male) and 310 (female)

^§^Hypertension was defined as systolic BTP ≥ 160 mmHg or using antihypertensive treatment on the regular basis

^§§^Diabetes mellitus at baseline was defined as f-glucose ≥ 6.7 or history of DM.

*SBP* systolic blood pressure, Mm-MAST Malmö-modified brief Michigan Alcohol Screening Test, *ESR* erythrocyte sedimentation rate

### Baseline HU

In total, 33,335 individuals (22,433 men and 10,902 women) were included and SU data were available in the vast majority of participants (22,368 men, 10,848 women). Altogether 2191 (9.8%) men and 164 (1.5%) women had HU (> 405 μmol/L) at baseline.

### Relation between baseline SU and other baseline characteristics

In men, SU at baseline correlated with higher BMI (*r* = 0.32), systolic blood pressure (*r* = 0.14), triglycerides (*r* = 0.25), Mm-MAST ≥2 (*p* < 0.001; Mann-Whitney test), lower estimated glomerular filtration rate (eGFR) (*r* = − 0.18), as well as with hypertension and CVD history (*p* < 0.001, Mann-Whitney test). Significant but weaker associations were seen between SU and total cholesterol (*r* = 0.11), Mm-MAST score (*r* = 0.08), fasting blood glucose (*r* = 0.04), erythrocyte sedimentation ratio (ESR) (*r* = 0.03), and history of one or several kidney stone attacks (*p* < 0.009 and *p* = 0.03, respectively; Mann-Whitney test).

In women, SU at baseline correlated with higher BMI (*r* = 0.38), age (*r* = 0.20), systolic blood pressure (r = 0.20), triglycerides (*r* = 0.30), lower eGFR (*r* = − 0.27), total cholesterol (*r* = 0.18), ESR (*r* = 0.17), fasting blood glucose (*r* = 0.13), and occurrence of hypertension, CVD and diabetes (*p* < 0.001; Mann-Whitney test).

Table 2 summarizes the baseline characteristics and comorbidities among participants in MPP stratified by sex and category of baseline SU levels (normal levels, i.e. ≤ 360; 361–405; and > 405 μmol /L).

**Table 2:** Baseline characteristics and comorbidities among participants in MPP stratified by sex and SU levels

	SU at baseline (μmol/L) (n of persons)	Age at baseline (years)	BMI (kg/m2)	Systolic blood pressure (pts not treated for hypertension # (mmHg)	eGFR (ml/min/1.73m^2^)	s-cholesterol (mmol/L)	s-triglycerides (mmol/L)	Hypertension n (%)	Sibling with hypertension; n (%)	CVD n (%)	DM n (%)	Kidney stone attacks ever; n (%)	Mm-MAST ≥ 2*^§^; n (%)	ESR (mm/h)	Smoking at baseline (n, %)
Men *n* = 22,433	Men with available SU at baseline														
	≤360 *n* = 16,824	43.6 (6.5)	24.2 (3.1)	129 (15.5)	80.0 (10.3)	5.6 (1.1)	1.4 (0.8)	2287 (13.6)	739 (4.4)	306 (1.8)	398 (2.4)	1461 (8.7)	4858 (28.9)	5.7 (6.0)	8688 (51.6)
	361–405 *n* = 3353	43.8 (6.6)	25.7 (3.3)	132 (16.2)	77.0 (10.5)	5.7 (1.2)	1.7(1.2)	712 (21.2)	181 (5.4)	83 (2.5)	90 (2.7)	305 (9.1)	1181(35.2)	5.7 (5.4)	1463 (43.6)
	> 405 *n* = 2191	44.5 (7.0)	26.9 (3.7)	134(16.6)	75.7 (11.0	5.8 (1.1)	2.1 (1.8)	662 (30.2)	177 (8.1)	84 (3.8)	86 (3.9)	203 (10.6)	867 (39.6)	6.4 (6.6)	861 (39.3)
Women *n* = 10,902	Women with available SU at baseline														
	≤360 n = 10,357	49.5 (7.5)	24.2 (4.0)	126 (17.6)	75.9 (10.8)	5.8 (1.1)	1.1 (0.5)	2130 (20.6)	1323 (12.8)	326 (3.1)	209 (2)	675 (6.5)	299 (2.9)	9.7 (7.5)	3636 (35.1)
	361–405 *n* = 327	53 (4.8)	29.0 (5.6)	136 (20.0)	70.2 (10.1)	6.4 (1.1)	1.7 (0.4)	173 (52.9)	56 (17.1)	23 (7.0)	27 (8.3)	33 (10.1)	**	13.4 (9.8)	105 (32.1)
	> 405 *n* = 164	53.1(4.6)	29.3 (5.3)	137 (18.2)	67.9 (14.6)	6.4 (1.1)	1.9 (1.1)	81 (49.4)	33 (20.1)	15 (9.1)	16 (9.8)	15 (9.1)	**	14.5(9.3)	53 (32.3)

*Mm-MAST = Malmö modified brief Michigan Alcohol Screening Test

**Calculations were not performed due to few observations

#Number of subjects with missing information were in different groups of SU were in men 13/2/2 and in women 434/11/5

§Number of subjects with missing information were in different groups of SU were in men 2521/314/201

*MPP* Malmö Preventive Project, *SU* serum urate, *BMI* body mass index, *eGFR* estimated glomerular filtration rate, *CVD* cardiovascular disease, *DM* diabetes mellitus, *Mm-MAST* Malmö-modified brief Michigan Alcohol Screening Test, *ESR* erythrocyte sedimentation rate

Both men and women with SU > 405 μmol/L had at baseline higher occurrence of hypertension, DM, CVD (*p* < 0.001, chi-squared test) and higher levels of BMI, total cholesterol, triglycerides, fasting blood glucose, systolic blood pressure, but lower levels of eGFR (*p* < 0.001, Mann-Whitney test) compared to those with SU levels ≤ 405 μmol/L.

A family history of hypertension among siblings was also significantly associated with SU > > 405 μmol/L (*p* < 0.001 and *p* = 0.017 in men and women, respectively).

Marital status did not differ statistically between individuals with SU > 405 μmol l/L and those with lower levels (data not shown).

### Lifestyle factors

#### Tobacco smoking

Men with HU were less often smokers at baseline and also a lower proportion of these patients had smoked for at least 10 years prior to baseline screening compared to those with SU levels ≤ 405 μmol/L (*p* = 0.001 and *p* = 0.004, respectively). Women with SU > 405 μmol/L did not differ significantly in smoking status compared to those with SU levels ≤ 405 μmol/L.

#### Alcohol consumption – Risk behavior

Men with SU > 405 μmol/L had significantly higher Mm-MAST score at baseline (mean score = 1.6 vs 1.3; *p* < 0.001 Mann-Whitney test) and higher occurrence of risk behavior for alcohol consumption, i.e. Mm-MAST ≥2 (*p* < 0.001, chi-squared test) compared to those with SU levels ≤ 405 μmol/L.

### Risk of developing gout

Overall, 1275 individuals (3.8%), i.e. 1014 men (4.5%) and 261 women (2.4%) of these middle-aged subjects developed incident gout during the follow up.

The absolute risks for incident gout, and the unadjusted and age-adjusted HRs, stratified by sex and baseline SU levels over the whole period of follow-up, are displayed in Table 3. Incidence rates and absolute risks increased substantially and progressively with increasing baseline SU levels. In the group with HU (> 405 μmol/L) at baseline the corresponding absolute risks were 13.3% (95% CI: 12.2–14.8) in men and 17.7% (95% CI: 12.4–24.6) in women, respectively.

**Table 3:** Incidence per 100,000 person years (p-yrs) at risk for developing incident gout in men and women by different levels of SU at baseline screening

No of individuals by sex and s-UA	SU at baseline (μmol/L)	Incident gout (n)	Person-years at risk	Incidence (95% CI) per 100,000 person-years at risk	Absolute risk (%)*
*Men (n = 22,368)*					
16,824	≤360	447	493,612	90 (82.2–99)	2.7 (2.4–2.9)
3353	361–405	227	95,408	238 (208.5–270.5)	6.8 (6.0–7.7)
2191	> 405	292	58,094	527.4 (471.4–588.1)	13.3 (12.0–14.8)
*Women (n = 10,902)*					
10,357	≤ 360	200	278,493	70.6 (61.2–81.1)	1.9 (1.7–2.2)
327	361–405	25	7655	340.9 (224.6–496)	7.6 (5.1–10.9)
164	> 405	29	3564	828.6 (566.7–1170)	17.7 (12.4–24.6)

*Mean follow-up (SD) for men 29.9 (8.7); 29.3 (8.9); and 27.9 (9.4); and for women 27.5 (7.3); 24.2 (7.5); and 23.5 (8.1) years

*SU levels were missing in two men and one woman, which explains the difference in number of incident gout

Since identifying a first-time gout diagnosis up to 1998 was based only on diagnosis given in inpatient care (probably more severe disease events) in contrast to the period after 1998 until end of study, when it was based on all visits to physician in the healthcare system, incidence rates and HRs are also presented by these two time periods (Table 4). In the first time period incidence rates and HRs (for men) were substantially lower compared to the second period. During both time periods there was a progressive increase in risk for being diagnosed with gout with higher baseline SU levels in both men and women. In the latter period this resulted in an age-adjusted increased risk in women of 4.4 (95% CI: 2.9–6.7; SU 361–405 vs ≤ 360 μmol/L) to 13.1 (95% CI: 8.8–19.4; SU > 405 vs ≤ 360 μmol/L) and in men of 2.7 (95% CI: 2.3–3.2; SU 361–405 vs ≤ 360) to 6.4 (95% CI: 5.6–7.5; SU > 405 vs ≤ 360 μmol/L). In this latter period there was a significant interaction between SU category and sex, with women having a significantly larger increase in HRs for incident gout compared to men with increasing SU levels categories (*p* < 0.001).

**Table 4:** Incidence per 100,000 person-years at risk and hazard ratios (HRs) for developing incident gout in men and women by different levels of urate at baseline healthcare screening. Results are presented separately for the two time periods; (a) baseline screening until 1998; and (b) from 1998 to end of study period

SU at baseline, μmol/L	Time period from baseline screening to 1998					Time period from 1998 to end of study				
	Incident gout (n)	Person-years at risk	Inc. (95% CI)*	HR unadjusted (95%CI)	HR age adjusted (95%CI)	Incident gout (n)	Person-years at risk	Inc. (95% CI)*	HR un-adjusted (95%CI)	HR age adjusted (95%CI)
*Men*										
≤ 360	8	322,368	2.5 (1.1–4.9)	1	1	447	188,039	237.7 (216.2–260.8)	1	1
361–405	7	63,617	11.0 (4.4–22.7)	4.4 (1.6–12.2)	4.4 (1.6–12.2)	227	35,336	642.4 (561.5–731.67)	2.7 (2.3–3.2)	2.7(2.3–3.2)
> 405	31	41,172	75.3 (51.2–106.9)	30.2 (13.9–65.8)	28.6 (13.1–62.3)	292	19,714	1481 (1316–1661)	6.5 (5.6–7.5)	6.4 (5.6–7.5)
*Women*										
≤ 360	1	143,968	0.7 (0.009–0.4)	n.a.	n.a.	200	137,282	145.7 (126.2–167.3)	1	1
361–405	2	4026	49.7 (5.6–179.3)	n.a.	n.a.	25	3735	669.3 (433.1–981.1)	4.7(3.1–7.2)	4.4 (2.9–6.7)
> 405	3	2048	146.5 (29.5–428)	n.a.	n.a.	29	1589	1825 (1222–2621)	14.0 (9.4–20.6)	13.1 (8.8–19.4)

*Per 100,000 person-years at risk

## Discussion

In the present prospective, observational study we report that the risk for incident gout over on average 28 years (mean follow-up 28.2 years and overall 936,826 person-years at risk) among middle-aged city residents was 3.8% (4.5% in men and 2.4% in women). The risk increased substantially with increasing levels of SU, and among individuals with HU the risk of being diagnosed with gout was 6- and 13-fold higher women in men and women, respectively. In this urban cohort with a mean age of 47 years at baseline, 10% of men and 1.5% of women had asymptomatic HU.

There are no European, and only few American [1, 15, 22] or non-Caucasian [8, 23], population-based cohort studies on the association between SU and risk of incident gout. Our study has the longest total follow-up of all such studies and a high participation rate. Only a Taiwanese study [23] is based on larger sample (but with a mean follow-up of only 7.2 years). Only the Framingham study [22] has a similar length of follow-up (based on 4427 subjects). Both these previous studies and ours have a balanced sex ratio and comparable mean age at baseline.

Comparison of risk for gout between these studies is possible only for subjects in the SU < 360 μmol/L category due to differences on categorization (i.e. quartiles or fixed other cutoff values) of SU in the different studies. In this subgroup, similar incidence rates as in our study where reported in a meta-analyses (including the above studies, 80 cases/100,000 person-years at risk) [24]. In the Framingham study, which had a similar length of follow-up as our study, the incidence rates were twice as high in men (195 cases/100,000 person-years at risk), but similar in women (103 cases/100,000 person-years at risk) compared to our results [22].

Another difference between studies relates to sex differences in the risk increase for incident gout with increasing baseline SU levels, where both the Taiwanese and the Framingham study showed a higher increase in men, whereas we showed the opposite. In analogy with this, the cumulative risk in those with baseline HU was comparable for men in the MPP and the Framingham study (15 vs 13%) but higher in the MPP for women (20 vs 6%).

Taken together, our results, in accordance with those of other studies, show that SU levels are strongly, and to a similar degree, associated with incident gout. Possible explanations for the modest difference in incidence and effect of sex on risk for gout with higher baseline SU between studies could be differences between populations with regard to genetics, comorbidities, lifestyle, or other exposures.

We also observed positive cross-sectional associations for both men and women between SU levels at baseline and systolic blood pressure and hypertension, BMI, fasting blood glucose, total cholesterol and triglyceride levels. SU levels were significantly higher in individuals with impaired kidney function determined by eGFR in both sexes. The lowest mean eGFR was observed in both men and women with the highest SU levels. These studies are largely in accordance with previous publications [24–29].

In a previously published meta-analysis, asymptomatic HU was associated with the development of hypertension independently of other traditional risk factors [24]. Furthermore, a recent Japanese study suggested that individuals with asymptomatic HU without other comorbidities were at an increased risk of developing cardiometabolic conditions such as hypertension, dyslipidemia, overweight/obesity, and chronic kidney disease, with a similar trend for development of DM [25]. On the other hand, several of these comorbidities, such as obesity [25, 26] and kidney function [27, 28], have convincingly been shown to predict the risk of both HU and gout [13]. In addition, an association between higher number of ideal cardiovascular health metrics (such as no smoking status, lower BMI, physiological fasting blood glucose level, healthy diet, better physical activity, normal blood pressure) and lower risk of asymptomatic HU was reported in a large longitudinal, population-based study from China [29]. Interestingly, this association was stronger in women than in men [29]. These results are in accordance with our observations and illustrate the close and complex relation between SU and these factors. Causality is not possible to conclude from epidemiological cohort studies as these and in studies using other techniques, i.e. Mendelian randomization, causality has been questioned for many of these associations [30, 31].

The association between alcohol overconsumption and gout is well-known [32, 33]. Since MPP was initiated as a screening program for identifying subjects where prevention through lifestyle changes (including alcohol use) might improve long-term health outcomes, a validated questionnaire, i.e. Mm-MAST [18–20] was used in order to identify alcohol risk consumption. We confirmed a significant positive correlation between alcohol risk consumption behavior (Mm-MAST) in men and HU at baseline.

Gout and HU are common in patients with chronic kidney disease although to what extent HU is the cause, consequence, or both, is still unclear [31, 34]. There is growing evidence that HU may potentially be involved in the initiation and progression of chronic kidney disease [28, 31, 34]. The inverse association between SU and eGFR at baseline was relatively strong in the MPP. In addition, men with increased levels of SU had significantly more often had nephrolitiasis. These results are in accordance with recently published data from a cohort study on approximately 240,000 healthy men where HU was independently associated with increased risk of kidney stones [35]. In addition, a recent Swedish study reported a 60% increased risk for kidney stones in patients with gout [36].

Another interesting observation in the present study is that SU levels at baseline correlated significantly with ESR, in particular in women. This association could possibly be confounded by BMI, which is known to be associated with low-grade inflammation [37]. On the other hand, other results support an association between SU and inflammation adjusting for BMI [37–40].

In the present study, we have deliberately focused on describing the risk for incident gout by different levels of SU and avoided analyzing the effect of other possible predictors, or adjusting for such. The temporal and causal relations between several of such factors and SU are complex and in some cases, as for example kidney function, possibly bi-directional. This is a challenge when attributing risk to individual factors/markers using traditional techniques for cohort analyses. Methods using, for example using Mendelian randomization based on genetic data, may be more suitable for this purpose [41]. However, there is a scarcity of large long-term studies determining the absolute risk for incident gout diagnosis in relation to levels of SU. Our report adds important information on this topic.

Other strengths of this study include its long follow-up time, high participation rate, the large sample size comprising over 33,000 healthy individuals from the same urban area, and the extent of data collection at baseline on several factors and comorbidities possibly associated with both HU and the development of gout.

There are also some important limitations of our study; first, no primary healthcare data were available from the time point of baseline screening and 1998. On the other hand, due to the expected increasing frequency of gout attacks in most patients over time and the documented low use of and adherence to ULT in southern Sweden [42], the vast majority of patients diagnosed with their first gout attack before 1998, are expected to seek healthcare help again and hence be identified as patients with gout at later time points. Second, we did not assess the proportion receiving ULT, but do not think this substantially affects our results, since there were no written initiatives to act upon notification of HU at the time for screening and the use of ULT has continued to be relatively low in this catchment area up through 2012 [42].

## Conclusions

Asymptomatic HU is common among healthy middle-aged individuals and is associated with an increased risk for incident gout in both sexes. In contrast to previous studies the risk to develop clinical gout at comparable SU values was found to be higher in women than in men. The close association between HU and several common comorbidities such as CVD, kidney disease, and hypertension, emphasizes the need to establish their causal relationships and whether ULT can decrease the risk for these comorbidities. To address these questions other types of designs such as randomized controlled trials (RCTs) and cohort studies using Mendelian randomization based on genetic markers of HU, will be crucial.

## Abbreviations

BMI: Body mass index
CI: Confidence interval
CVD: Cardiovascular disease
DM: Diabetes mellitus
eGFR: Estimated glomerular filtration rate
ESR: Erythrocyte sedimentation ratio
HR: Hazard ratio
HU: Hyperuricemia
ICD10: International Classification of Diseases version 10
Mm-MAST: Malmö-modified brief Michigan Alcohol Screening Test
MPP: Malmö Preventive Project
SHR: Skåne Healthcare Register
SU: Serum urate level
ULT: Urate-lowering therapy
